# Constructing community in the Neolithic of southern Jordan: Quotidian practice in communal architecture

**DOI:** 10.1371/journal.pone.0193712

**Published:** 2018-06-13

**Authors:** Cheryl A. Makarewicz, Bill Finlayson

**Affiliations:** 1 Institute for Prehistoric and Protohistoric Archaeology, Christian-Albrechts-Universität zu Kiel, Johanna-Mestorf Strasse 2–6, Kiel, Germany; 2 Council for British Research in the Levant, 10 Carlton House Terrace, London, United Kingdom; University at Buffalo - The State University of New York, UNITED STATES

## Abstract

The emergence of food production during the earliest Neolithic of the Near East was accompanied by profound changes in the ways in which societies were organized. Elaborate and multi-stage mortuary practices involving the removal, caching, and plastering of symbolically charged skulls are thought to have played an important role in cross-cutting household lines to integrate communities and maintain social cohesion during the late tenth to ninth millennium cal BP, particularly in Middle Pre-Pottery Neolithic B settlements located in the southern Levant. While the ritual and mortuary activities associated with skull manipulation were dramatic and high impact occasions that drew people and households together, it is likely they were highly episodic and, consequently, attendant community cohesion susceptible to decay over time. Recent research in southern Jordan, where skull plastering was not practiced as seen elsewhere in the southern Levant, has revealed that non-residential building structures were a common feature of early Pre-Pottery Neolithic settlements. Renewed excavations at Beidha, a Middle PPNB settlement located in the Shara’a mountains, have revealed a large, easily accessible communal structure that provided a focal point in which mundane, informal daily activities could regularly take place. The routine and repeated interactions fostered by such non-domestic structures facilitated highly durable modes of community cohesion and was part of a temporally deep ethos of community that first emerged a thousand years earlier when people first began to experiment with plant cultivation. It appears that in southern Jordan, a distinctive social cohesion pathway developed that engaged community daily practice within non-residential buildings to maintain and strengthen social structures, rather than occasional and dramatic ritual and mortuary practices used elsewhere in the southern Levant.

## Introduction

The transition from hunting and gathering to food production in the Near East unfolded over twelve thousand years ago at various tempos and along diverse developmental trajectories that were shaped by local resource availability, cultural traditions, and knowledge sets [1, 2, 3]. The emergence of new subsistence strategies focused on cereal cultivation during the early Pre-Pottery Neolithic appears to have been accompanied by profound changes in social organization with settlements structured by a community-based ethos. By the late eighth millennium cal BC when massive agro-pastoralist villages supporting large populations appeared, the importance of community-based structuring elements declined dramatically and were replaced by increasingly autonomous corporate households that owned resources and served as the locus of social and political power within the community [4, 5, 6].

Identifying shifts in Pre-Pottery Neolithic social organization has, in general, been inferred from the architectural record through scrutiny of the overall scale, configuration and internal composition of individual buildings, the spatial relationship between buildings, as well as examination of the burial record. Based on the ubiquity of single-room circular and sub-circular residential structures, often described as ‘houses’, present in Pre-Pottery Neolithic A (PPNA) settlements, the earliest food-producing societies in the Near East are generally understood as organized along independent nuclear family households that served as the primary unit of production and consumption [7, 8, 9]. However, other organizing principles also structured these communities as indicated by diversity in PPNA building forms that is a common feature of PPNA settlements but, surprisingly, neglected in discussions on early Neolithic social organization. Distinctive in form between and within settlements, these structures varied in their shape, size, and internal configuration, which often included unusual and sometimes elaborate internal architectural features including platforms, benches, and stone cists, as well as interior treatment of floors and walls (e.g. plastering and painting). The wide variety of these architectural forms, in addition to the regular construction of buildings for specific, non-residential purposes, may indicate that the nuclear family household was less important at this early stage of community organization than previously thought. For example, various PPNA buildings have been interpreted as designed for use by community members, including the *bâtiments communautaires* at Jerf el-Ahmar used for both shared storage and gatherings [10], a large granary for collective storage of cereal at Dhra’ [11], the large benched structure at Wadi Fayan 16 that served as an arena for both mundane and more formalized performances [12], and the likely communal mortuary structure at el-Hemmeh [13], together share a non-residential character and appear to have played a crucial role organizing early Neolithic societies above the nuclear family by providing a focal point for conducting formalized ritual activities as well as routine and mundane social interactions, performing tasks, and storing food resources [14, 15]. Truly monumental constructions found in other regions of the Near East, such as the tower at Jericho [16] and the T-shaped pillar structures at Göbekli Tepe [17], while important features of early Neolithic landscapes that orchestrated communication and interaction between far-flung communities [18, 19], appear to have had little, if any, role in structuring the daily social lives of PPNA community members in some regions, such as southern Jordan.

Subsequently, during the Pre-Pottery Neolithic B (PPNB, 8,500–6,700 cal BC), the development of rectilinear and partitioned buildings has been interpreted to indicate the replacement of the nuclear family by the extended family household, which functioned as a corporate entity, in structuring Neolithic societies [6]. Unlike PPNA settlements, where various forms of communal architecture were common features of the built environment, the use of such architecture for communal purposes, whether quotidian or ritual in orientation, declined precipitously during the PPNB. Relatively few communal buildings have been so far identified in southern Levantine settlements dating to the later PPNB, limited to LPPNB the ‘temple’ and ‘shrine’ structures at ‘Ain Ghazal located in northern Jordan [19], and also the large (up to 105m^2^) Buildings 8 and 9 in in the final MPPNB phases at Beidha located ca. 250 km to the south [20]. These buildings may have served as venues for supra-household decision making and ceremonial activities that helped maintain and integrate communities that were otherwise being pulled apart by increasingly autonomous households [6,20].

### Pre-Pottery Neolithic societies in southern Jordan

Throughout the early Pre-Pottery Neolithic, southern Jordan was recognizably different from other areas within the southern Levant, including northern Jordan, the Galilee, and the west bank of the Jordan river, distinguished by locally specific subsistence strategies and different architectural forms in settlements [14]. Pre-domestication cultivation of barley and use of legumes and figs were important in early Pre-Pottery Neolithic subsistence economies throughout the southern Levant [21], but animal exploitation strategies differed across the region with intensive hunting of goats in southern Jordan rather than gazelle which were common prey elsewhere in the region [22]. For PPNA settlements located west of the Jordan Valley, buildings were circular or elliptical in shape, lacked internal partitions and were physically separated from each other, while contemporaneous settlements in southern Jordan exhibit a much more diverse architecture, frequently agglutinative, that included semi-subterranean and free standing circular, elliptical, irregular, and squared forms constructed out of stone, pisé, or a combination of both materials (12, 23). These buildings were also internally complex and contained features including partitions, benches, raised platforms, and molded hearths as well as other eclectic elements unique to particular structures. Furthermore, wall configurations and internal features were typically modified throughout the use life of buildings. Later, during the Middle PPNB, architecture in southern Jordan initially maintained a circular shape, unlike other regions of the southern Levant where domestic architecture took on a rectilinear plan [23, 24], a shift that was completed in southern Jordan by the Late PPNB.

While some burial and associated mortuary rituals practiced in southern Jordan were shared with the rest of the southern Levant, in particular the PPNA practice of interring individuals on the side in a flexed position under house floors, within destruction/abandonment layers in buildings, and in outdoor spaces within accumulating occupation deposits, there was considerably more diversity in burial practices within southern Jordan within and between individual sites. In addition to arranging bodies in a flexed position, interred individuals were laid out in a fully extended position, on the back with the knees lying to the side, and in a fully upright seated position [25, 26]. Burials at WF16, el-Hemmeh, and ‘Dhra were regularly marked with shaped stones and pisé platforms, while at el-Hemmeh, numerous individuals were within purpose-built stone cists situated within a single building [25]. In addition, while, no secondary burials have been identified at el-Hemmeh or Dhra, several have been uncovered at WF16 and Sharara which include communal interments consisting of skull elements representing multiple individuals, fragments of skulls from multiple individuals but associated with a single primary burial, and multiple skulls arranged with collections of bones from multiple individuals. Modification of human bone, in particular cranial parts, was also practiced at WF16, and is represented by a painted cranial vault belonging to a complete skeleton and also a drilled and polished cranial fragment [26].

Subsequently during the Middle PPNB, mortuary practices in southern Jordan emphasized burial of entire individuals placed within specific buildings, for example on the floor as seen in Building 41 at Beidha, and at Shkarat Msaied, below the floor in Building F, which appears to have served as a mortuary structure, which contains over 67 primary and secondary burials. Perhaps most striking, Middle PPNB mortuary rites in southern Jordan did not include skull plastering as practiced elsewhere in the southern Levant [27, 28].

### Communal architecture in Southern Jordan

**Fig 1:**
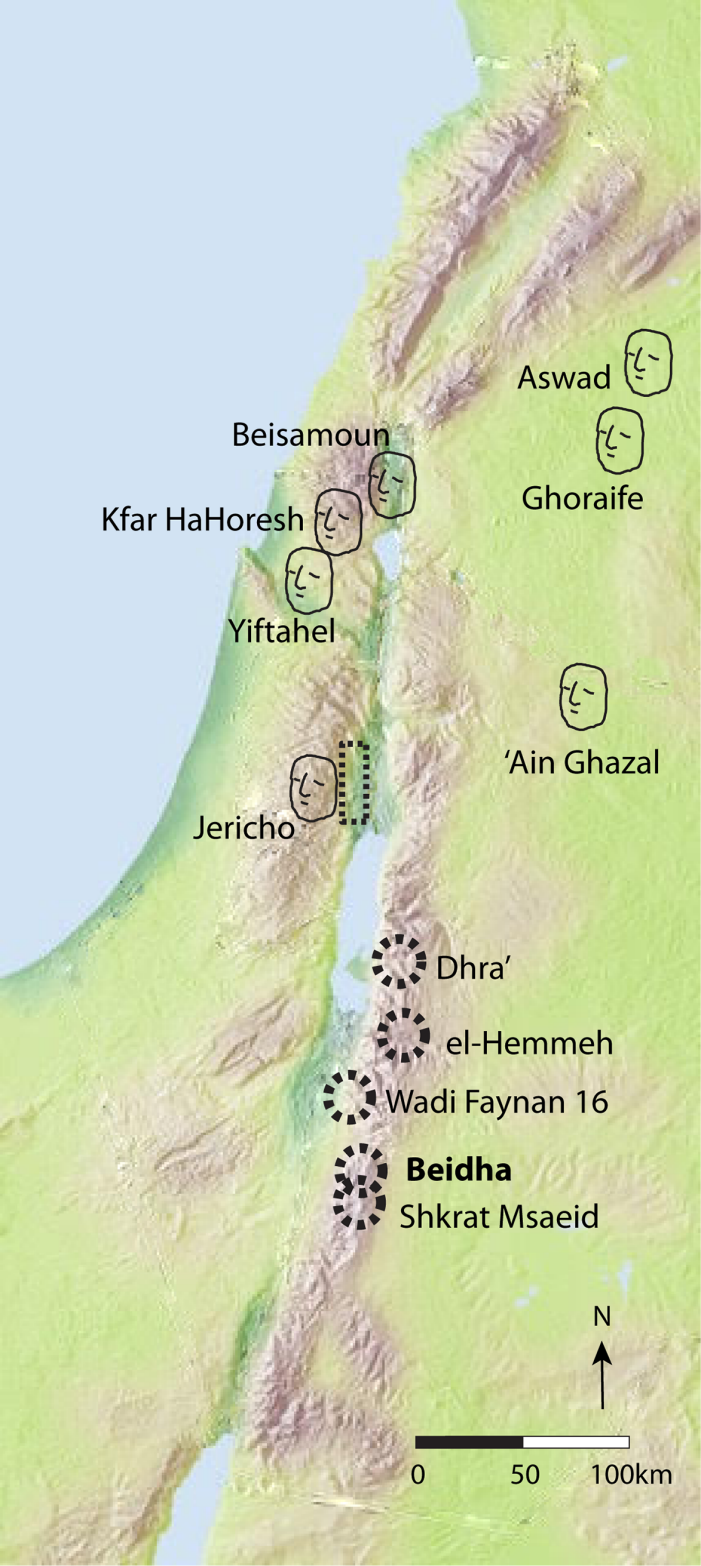
Location of MPPNB Beidha and other southern Levantine PPN sites. Dashed circles indicate PPN settlements with communal architecture. Face symbols indicate MPPNB settlements where skull plastering was practiced. Dashed box indicates monumental architecture.

Architecturally distinct non-residential structures also regularly featured in Pre-Pottery Neolithic settlements located in southern Jordan (Fig 1) [14, 23]. Such structures, variable in architectural form, were in use by the 12th millennium cal BP during the PPNA and served multiple functions variously associated with food production, performance and celebration, and mortuary practice. At both Dhra’ and WF16, substantial sub-circular pisé structures containing notched upright stones that supported raised floors and, at Dhra’, yielded cereal phytoliths, are best interpreted as granaries [11, 12]. The construction of separate granary buildings at both Dhra’ and WF16 suggests storage was intentionally made open and visible to ensure sharing and communal use of cereal grains [11, 12]. Maintaining community was also important in death, suggested by the large (ca. 8m in length) semi-circular stone structure at el-Hemmeh containing several clusters of human burials arranged in upright seated or semi-flexed positions on their side and placed in semi-subterranean stone cists demarcated by basalt and limestone markers [13, 25]. This mortuary structure is demarcated from all neighbouring buildings by its semi-circular stone wall and, although it appears to have been open to the west, its western perimeter would have been marked by the hillslope that defines this edge of the site. However, the structure would have been equally accessibly to all members of the community through this open aspect [25].

Architecture also embodied the closely intertwined nature of profane and symbolic activities in PPNA lifeways. Mundane activities practiced on a daily basis involving food processing and crafting of everyday tools and implements were often conducted in the same building as infrequently-held events imbued with ritual and symbolic meaning. This is perhaps best exemplified by the impressively large, over 20m in diameter, pisé and mud plaster structure at WF16 which contains multiple, distinct internal features associated with different types of activities. Cup-hole mortars set in raised portions of the interior floor surface indicate food processing activities were undertaken in a visible communal setting, while symmetrical tiers of benches rising above the interior floor suggest the space was used for celebrations and performances for sizeable audiences [12]. While the precise activities and communication pathways facilitated by the shared use of large granaries, mortuary structures, as well as production and performance arenas accessible to the community above individual families remain unclear, the ubiquity of such communal architecture in PPNA settlements strongly suggests that the social systems of increasingly large and settled PPNA communities were supported by the propagation and maintenance of community-oriented activities.

The use of communal buildings in southern Jordan continued well into the 10th millennium BP, evidenced in particular by unusually large circular buildings present at Shkarat Msaied and Beidha [27, 20]. Building F at Shkarat Msaiad is substantially larger than, but otherwise architecturally very similar to, the other buildings that compose the settlement, but is notably distinct in that it contains a large number of human burials thought to represent the entire community [27]. At the nearby settlement of Beidha, Building 37, which had only been partially excavated by Kirkbride during the 1960s, was interpreted as a communal building on the basis of its large size [20]. Notably, both domestic and non-residential MPPNB architecture in southern Jordan maintained a circular shape well into the Middle PPNB in contrast to rest of the southern Levant where domestic architecture took on a rectilinear plan [24, 20]. The precise phasing of the group of sub-circular structures located by Kirkbride to the east of the main site at Beidha, and identified as having a ritual role, is unknown, but it is possible that these represent a late and anachronistic use of circular architecture for non-domestic purposes, as appears to have been the case for circular architecture in Late PPNB Ain Ghazal in northern Jordan [24,20].

By the end of the 9th millennium cal BP during the Late Pre-Pottery Neolithic B (LPPNB), the construction and use of communal buildings had ceased in southern Jordan. This development coincided with a dramatic increase in settlement size from *ca*. 2 to 14 hectares, a radical change in building form characterized by densely packed agglutinative architecture containing both larger rooms and numerous small (2-4m^2^) rectilinear compartments linked internally by doorways, windows, and stairways, as well as a major shift in food production strategies to include for the first time intensive herding of domesticated sheep and goats alongside domesticated barley and wheat agriculture [21, 22, 28, 29, 30]. The new reliance on plant and animal domesticates, use of architectural forms that increased storage capacity for agricultural output while simultaneously concealing such stores from neighbors, and the absence of non-residential buildings in LPPNB settlements suggests the emergence of new socio-economic frameworks that accentuated individual or household property and privacy [31, 32].

Although communal buildings were a highly visible and distinctive component of early Neolithic settlements in southern Jordan and other regions of the Near East, they have been relegated to a lesser role in facilitating social interaction and integration in favor of the widely-known MPPNB mortuary practices of skull removal and plastering, thought to have been an essential social mechanism that both reinforced group cohesion and supported the development of multi-generational lineages [33, 34, 35]. While skull removal was practiced at PPNA WF16 [26], although not at Dhra’ or el-Hemmeh, and skulls were collected at MPPNB Shkarat Msaied in a stone cist located in Building F [27], the MPPNB practice of skull plastering was not undertaken in southern Jordan suggesting that alternative means of social integration were in operation in this region (Fig 1). Through renewed excavations at Beidha, a MPPNB settlement located in the Shara’a mountains in the Petra region of southern Jordan, we more closely evaluate the role of communal buildings in structuring social organization during the MPPNB. We focus on Building 37, an unusually large structure associated with the earliest occupation of the site, in order to further investigate the evolving role of communal buildings in early Neolithic societies and how they may have facilitated community integration within a context of increasingly autonomous households.

## Materials and methods

**Fig 2:**
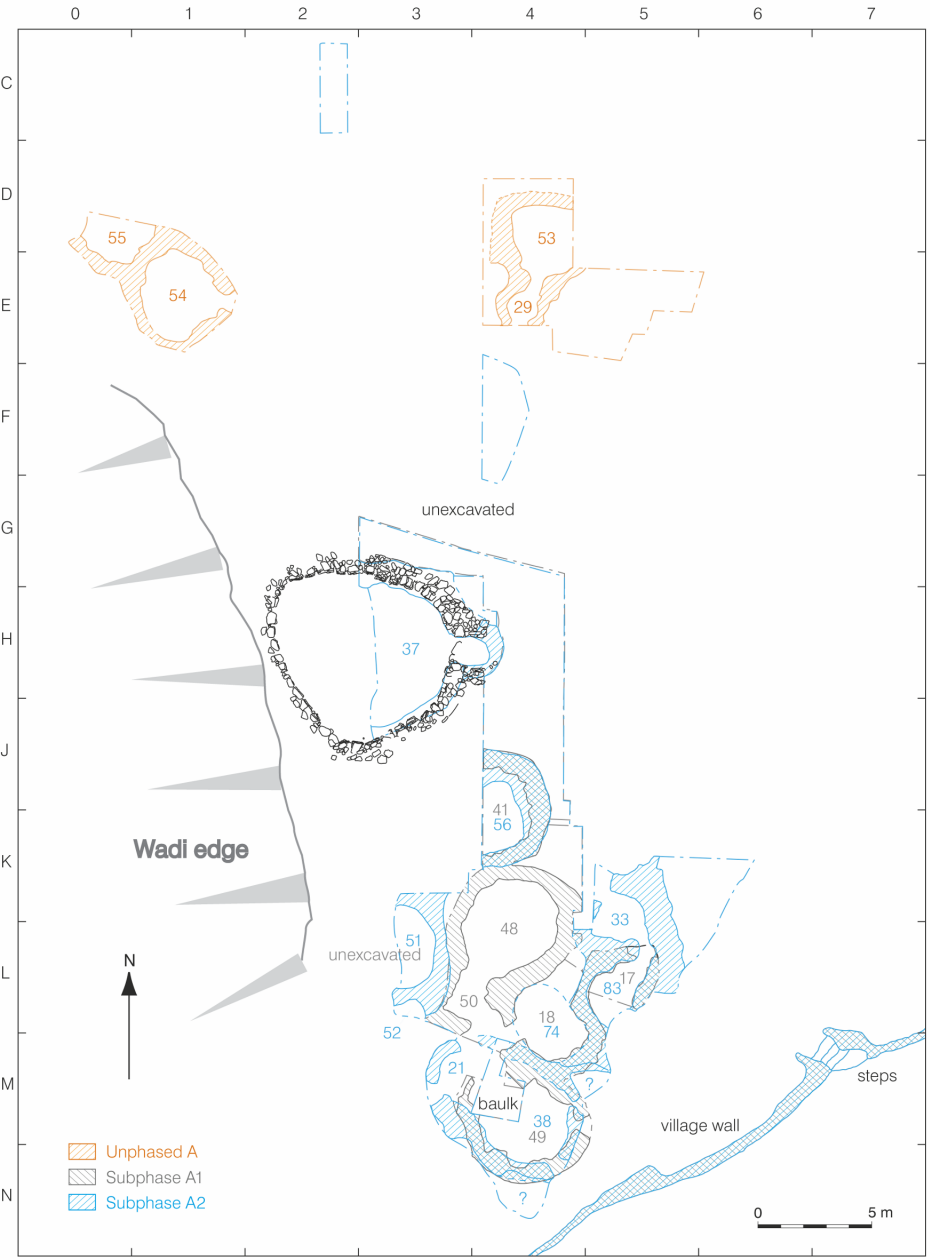
Phase A MPPNB architecture at Beidha. Spatial relationship of Building 37 to Phase A1 and A2 domestic structures.

The Middle PPNB settlement of Beidha is situated on small terrace overlooking a small wadi system that today supports dry farming (Fig 2). Beidha was excavated during eight seasons of work between 1958 and 1983 during which time ca. 1,500 sq m of Neolithic deposits were exposed. Recent re-analysis of the stratigraphy distinguished three phases of occupation: the earliest (A) identified by the presence of circular stone buildings, followed by Phase B consisting of sub-rectangular stone buildings, and finally the latest occupation (C) defined by stone corridor buildings [20]. New excavations were conducted during 2014 with permission from the Jordanian Department of Antiquities to investigate one large Phase A structure, Building 37.

Based on previous and new radiocarbon determinations obtained from Phase A and contexts associated with Building 37, this structure was in use during the early MPPNB (ca. 8000 cal BC). Radiocarbon determinations associated with Building 37 include two charcoal samples, one obtained from a layer of collapsed structural material (141) (Poz 67013, evergreen oak, 8760± 50 uncal BP, 8166–7602 Cal BC at 95.4%) and the other from a layer of compacted, probably trampled material (109) (Poz 67011, *Pistacia* sp., 8770± 50 uncal BP, 8170–7609 Cal BC at 95.4%). Both samples were retrieved from above the burnt roof material and from contexts possibly deriving from the collapse of the wall. The third charcoal sample (Poz 67010, *Quercus* sp., 8720± 50 uncal BP, 7939–7601 Cal BC at 95.4%) is derived from the sterile sand (103, described below) underlying the Building 37 floor. These three samples yielded similar radiocarbon determinations dating to the early part of the MPPNB (8170–7601 cal BC, 95.4% probability), while the fourth date obtained from a piece of oak charcoal (Poz 67014, 8860± 50 uncal BP, 8224–7795 cal BC at 95.4%) retrieved from the fill of one of the postholes (126) located below the stone sub-floor sand was fractionally older, but still MPPNB in date. All dates are modeled in OxCal v.4.2, using IntCal13 calibration curve [36, 37].

Building 37 is part of the earliest (Phase A) occupation of Beidha which is broadly dated to the early MPPNB and divided into two sub-phases A1 and A2, although four phase A buildings cannot be separated into either sub-phase (Fig 1; [20]). Phase A architecture, previously described as ‘domestic dwellings’ [20], consists of circular semi-subterranean stone structures replete with post-sockets set along the interior face and, in most cases, a single central posthole. Phase A circular buildings range in size between 2m^2^ to 14m^2^ in area, with the interior floor area ranging between 1.7 m^2^ and 5.0 m^2^ for ‘small’ buildings (mean = 3.9 m^2^) and 7.4m^2^ to 14.9m^2^ for ‘medium’ buildings (mean 10.6 m^2^) [38]. Phase A buildings were laid out in tight clusters, frequently shared walls, and often had more than one entrance, although remodeling of the wall circuits and blocking of doorways suggests that only a single entrance may have been in use at any one time.

The six subphase A1 buildings lacked internal features other than a posthole, but contained rich inventories of in-situ material culture, including bitumen baskets, wooden bowls and boxes, bone tools, stone bowls, querns, pestles, groundstone axes, and shell beads set on floors [20]. Some of the eight sub-phase A2 domestic buildings lacked a central posthole hole, but several contained a single feature (e.g. a hearth set into a plaster floor or a raised stone platform); smaller in situ material inventories were also present on floors.

Phase A architecture was regularly repaired and modified, with new floors laid down, walls buttressed, and overall building configurations modified during their occupation. A2 constructions frequently re-used phase A1 building walls and it is possible that two subphase A1 buildings were continuously used into subphase A2, increasing the architectural density of the cluster of domestic structures. Several of the A1 buildings and two-thirds of the A2 buildings were destroyed by fire, evidenced by collapsed roofs containing considerable carbonized material including central posts, roof beams, and heavily burned roof clay imprinted with beams and reeds. Previous interpretations have attributed the burning to a single catastrophic fire that precipitated large-scale abandonment of the settlement [20]. However, recent experimental work at Beidha indicates that burning of Phase A type buildings requires a significant amount of effort to ignite and maintain, suggesting that firing buildings was an intentional activity and part of the formal process of closing structures [39].

The only human interments associated with Phase A are from subphase A1 Building 41, only a quarter of which was excavated. Seven individuals were interred in midden deposits that accumulated within the abandoned building as part of a succession of separate burial events, some of which disturbed earlier interments. Young adults, young children, and newborns all received burial treatment and were arranged in flexed and semi-flexed positions (McClelland unpublished report). Some individuals were placed on flat stone slabs (e.g. infant burials 3 and 7) or their head propped up on a ‘pillow’ stone (e.g. burial 5, five year old child). Notably, all buried individuals retained their cranium.

### Original excavations of Building 37

In addition to the domestic buildings identified in phase A, an unusually large, circular structure, Building 37, was partially uncovered during the excavations conducted during the 1950s and 60s. Located on the westernmost edge of the site alongside the edge of a wadi that runs along the settlement (Fig 2), Building 37 was originally described as a large circular building ca. 6.2m in diameter constructed of large unmodified cobbles with the interior stone face lined with vertical post-sockets inset into the wall. At the time, it was thought that half of the structure had been excavated based on the estimated interior curvature of the Building 37 wall. Building 37 was interpreted as a structure that stood slightly apart from all other buildings in the settlement based on the absence of architecture abutting the exterior eastern and southern portions of the wall face.

During the Kirkbride excavations, a passage entrance that extended beyond the perimeter of the exterior of the primary Building 37 wall was identified on the east side of the structure (Fig 2). Two threshold stones were also uncovered, evidenced in the original excavation photos although not discussed in the original excavation report. This entryway was later modified and turned into a small alcove which appears to have differed in construction from the rest of Building 37 according to Kirkbride’s original photographs, distinguished by small cobbles in the one-course wide wall construction and absence of wall-post-sockets. Although it is unknown if this alcove wall was removed during original excavations or collapsed later on after exposure, the absence of any remaining traces of the alcove wall suggests it was not bonded to the original construction and the alcove was a later installation that blocked the passage entryway. The alcove was later partially blocked with additional stone cobbles.

Kirkbride’s excavation of Building 37 also revealed a floor composed of tightly packed irregularly shaped stones with at least one flat surface. The spaces between flooring stones were originally described as filled with gravel and no mention of a clay or plaster surface covering the flooring stones, although there is somewhat contradicting information reported mentioning the presence of a ‘thin layer of clayey occupation debris’ directly on the floor ([20]: 37). The original excavations also identified a stone lined posthole, ~ 50cm in diameter with a base ~ 75cm below the floor, in Building 37 and plaster of an unknown material preserved around the post itself. A series of large stone slabs sitting directly above the floor was also identified. Protruding from the western section profile, these slabs were interpreted as a platform that had collapsed onto the floor. One slab was originally described as being quite large (1.5 x 2.0m) size, but pictures and horizontal plans from the original excavations indicate a much smaller stone 1m x 50cm in size that remained partially covered by unexcavated deposits ([20]: 265–266).

### Renewed excavations of Building 37

In light of recent research indicating the prominent role of communal buildings in structuring the earliest Neolithic societies in southern Jordan, we renewed excavations of the Building 37 in order to better 1) delineate the overall structural form and internal features, 2) document the construction history and 3) establish the chronology of construction and use of the building. Beidha is a part of the Petra World Heritage site, and the extent of the new excavations was tightly controlled in order to minimize its impact on remaining deposits and maintain the site and building for display. Renewed excavations revealed that Building 37 is distinguished from other contemporaneous phase A architecture by the manner in which the a) building site was prepared for construction, b) way in which Building 37 stood slightly apart from the other tightly grouped buildings, c) construction of a floor designed for heavy use as well as evidence for intensive use of the floor, d) presence of a group of shaped stone slabs in the building (although this is not unique–Building 54, for example, has a series of slabs set into its floor), and e) its large size. At the same time, Building 37 shares numerous features with other Phase A buildings, including a lack of interior divisions, passage entryways, and later blockage of entryways with stone cobbles.

The new excavations reveal that the site for Building 37 had been carefully prepared before the building was constructed. Although not explicitly mentioned by Kirkbride, examination of photographs suggests a foundation pit was excavated for Building 37 indicated by the difference in height between the stone surface in the building and the contemporaneous ground surface visible through the original entrance (pre-alcove) located on the east side of building of the interior stone floor ([20]: plate 173). The new excavations indicate that the pit reached, and may have truncated, an earlier occupation layer represented by post- and stake-holes cut into a layer of compact sand with a high ash content (Context 119). This occupation layer was overlain by ca. 10 cm of sterile sand (Context 103) which apparently functioned as lining of the presumed foundation pit prior to the construction of the Building 37 wall and floor. Outside of forming a level surface at the base of the pit, the sterile sand would not have provided any additional structural benefits to the building.

**Fig 3:**
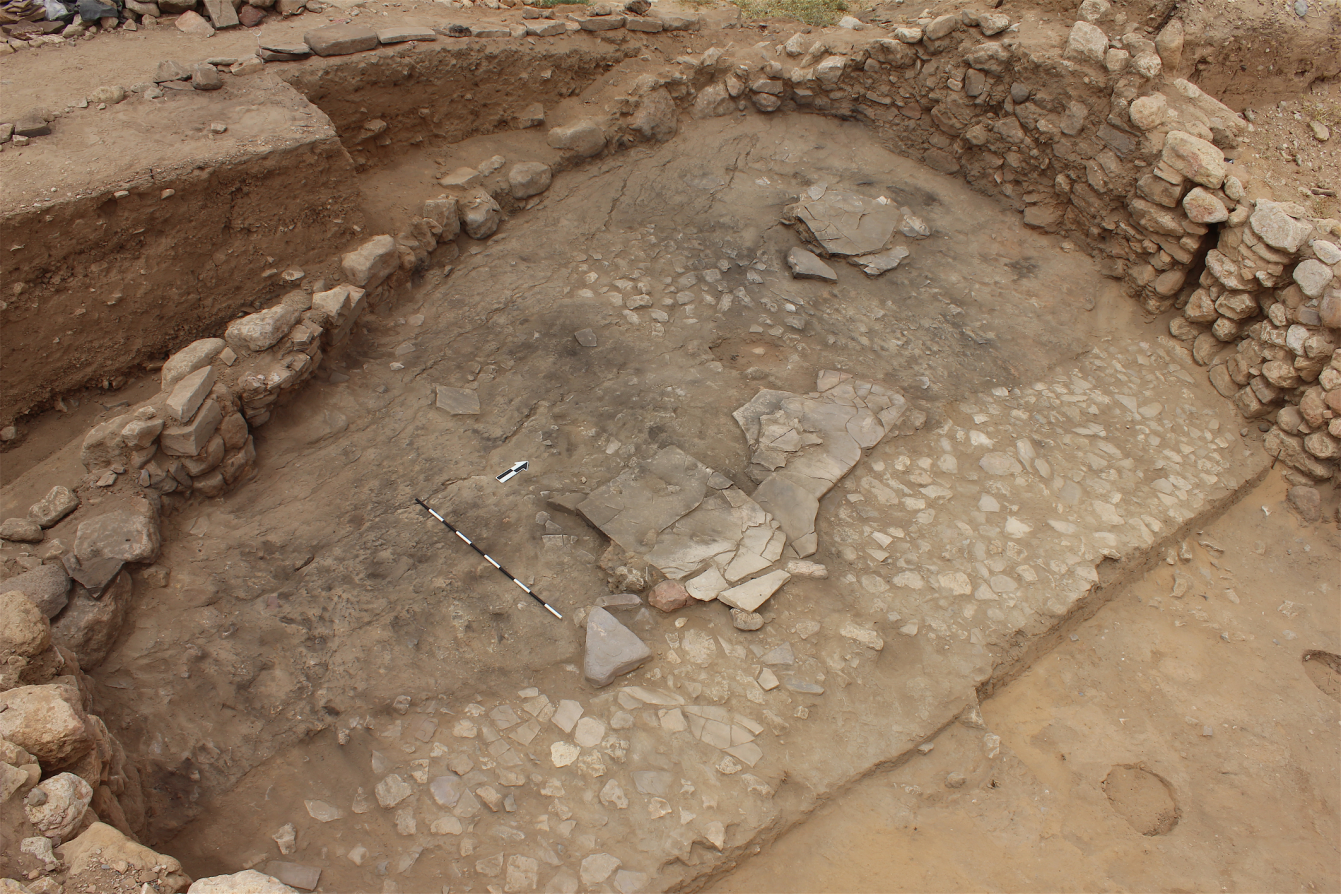
Building 37 floor surface and subfloor. View of Building 37 clay floor laid over tightly packed smaller stones. Bare patches located in the northern quadrant of the clay floor reveal the foundation stones underneath and suggest the clay surface was worn through from heavy use.

An unusually well-constructed and robust floor designed for heavy use was installed in Building 37 with the robust circular wall identified during Kirkbride’s excavation, here named Wall 1. The new excavations revealed that this floor (Context 149) consisted of a 1cm thick, extremely compact sterile clay material worked in between and over tightly packed small, flat stones (Context 148) which mortared the stones together to create a smooth surface (Fig 3). The flat stones, originally interpreted by Kirkbride as the floor surface [40], instead served as a sturdy foundation for the clay that formed the actual floor surface. It is clear that the extremely compact clay is not occupation debris as described in the original report as it contained no cultural material or ash. Patches of gravel (context 157), also identified by Kirkbride, were also placed on some areas of the stone surface, probably to level uneven areas of the stone sub-floor before the clay surface was laid down.

This robust floor design, not present in any other phase A structures which instead contained thin plastered floors, appears to have accurately anticipated intensity of use. Notably, the clay floor surface was not present in some areas, particularly in the northeastern portion of the building, leaving the foundation stones (148) exposed (Fig 3). The clay surface thinned out around these bare patches of stone surface, suggesting these patches were places where the floor wore out from repeated use.

Renewed excavations revealed a second substantial post-hole (context 150) inside Building 37, 35cm in diameter and 40cm deep, well-lined with flat elongated stones firmly wedged in with chocking stones; the carbonized remains of an upright timber were also identified in this posthole. Building 37 was apparently sufficiently large that its roof required two posts to hold it up, contrasting with the single post-holes found in other Phase A buildings.

**Fig 4:**
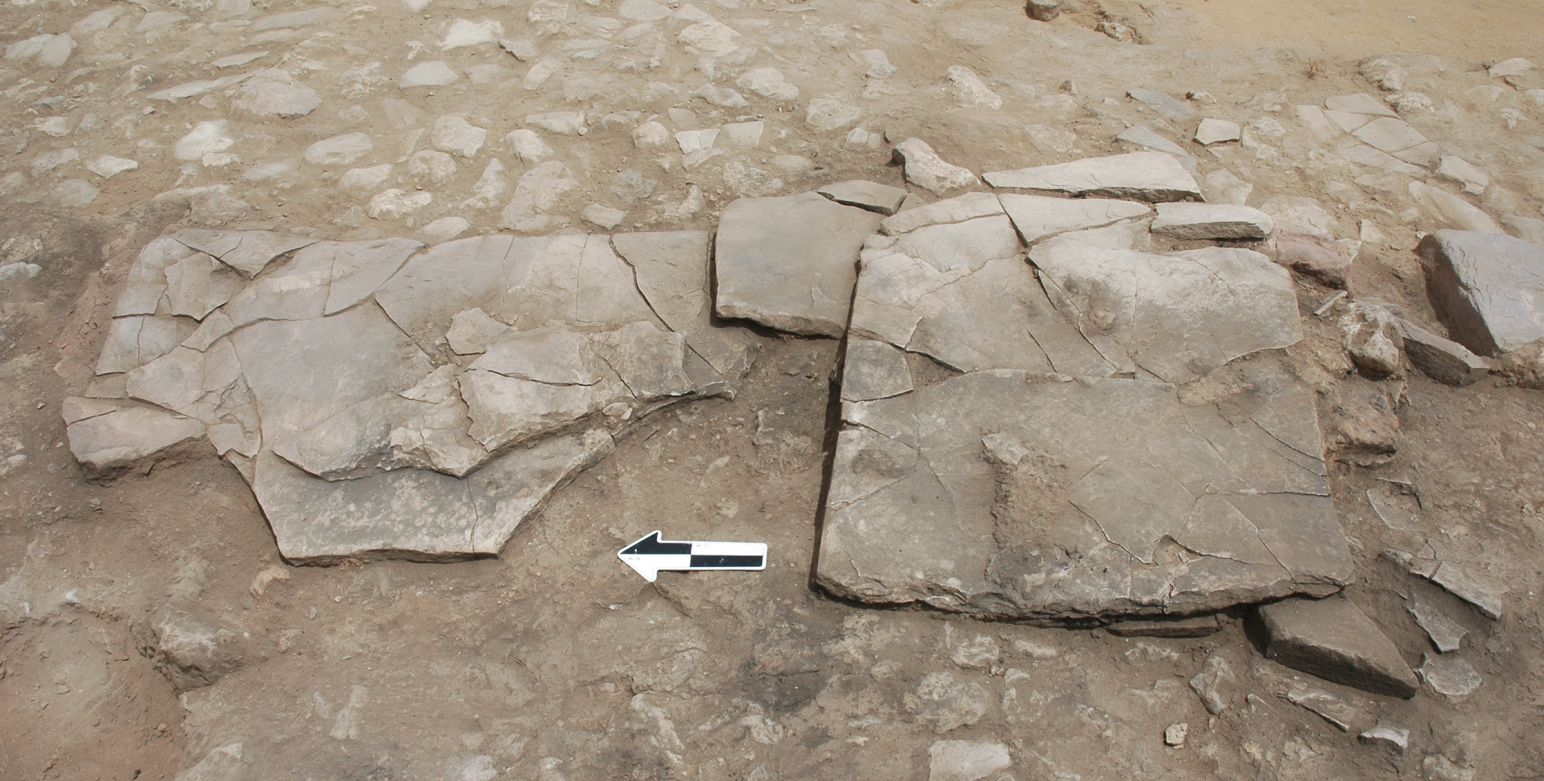
Enigmatic stone slabs in Building 37. Stone slabs, the easternmost portion of which were identified in the original Kirkbride excavations, sitting on top of the clay floor surface.

No internal architectural features other than the post-holes were present in Building 37, but other, possibly portable, features were identified. The stone platform initially exposed in the 1960s was fully excavated revealing two large slabs (context 153 and 154) and a smaller slab (context 159), which lay between the larger stones, together forming a tight, overlapping group (Fig 4). All the slabs are made of local sandstone and are ca. 2cm thick. One slab (context 153) displays a series of incisions cut along its northern edge, almost identical to the incisions found on a stone slab at the nearby MPPNB site of Shkarat Msaied, there used as part of a stone burial cist in Building F ([27], 70, and Fig 2.48 p 365). The western end of the same slab had been flaked to produce a straight edge, while its eastern end was shaped in a convex form, making the slab similar in overall shape to the slab from Shkarat Msaied described as a stele ([27], Fig 2.52, p 368). Slab (context 154) had been flaked on its eastern end to produce a straight edge while its opposed, western end had been flaked into the shape of a truncated triangle. The smaller slab (context 159) had been shaped to produce the same form as (context 154), but lay with its straight edge to the west.

It is highly unlikely that these slabs in Building 37 functioned as a platform as initially thought during the original excavations. A very thin (<1cm) layer of clean sand (Context 158) was identified between the surface of clay floor 148 and the stone slabs, and, a few stones were wedged under some slabs. This suggests the slabs were laid carefully onto the clay floor with some protection and stability created by the sand deposit and wedging stones. The careful shaping of the slabs and the presence of a straight edge on all three of them may indicate they were originally intended to stand upright, perhaps to form a small feature such as the cist at Shkarat Msaied building F. The slabs are too thin to have stood on their own, and equally, would have been too fragile to have formed a functional platform surface. The slabs would have been relatively portable, and easy to support with clay buttressing, or even bound together with rope. A concentration of baked clay above and around the slabs was identified during excavation, but it was difficult to discern if this was detritus from roof collapse or was directly associated with the stones. It may be that the slabs were intentionally, and carefully, placed on the floor of the building before it was burnt down. While it would be facile to assume a ritual role for such shaped slabs, their similarity in material, shaping, and cut marks with slabs from Shkarat Msaied employed in mortuary architecture, suggests that they had some symbolic connotation.

An isolated stone slab (context 152) was also found near the newly excavated post-hole (context 150), which was situated on a thin layer of ashy material (context 146). Below this ash, another slab of approximately the same size and shape as stone 152 embedded in the clay floor (149) was identified. The upper slab appears to have been put in place to mark the location of the lower slab very soon after the roof burning took place, possibly even during the course of roof collapse, as it was buried below the main layer of charred timbers.

**Fig 5:**
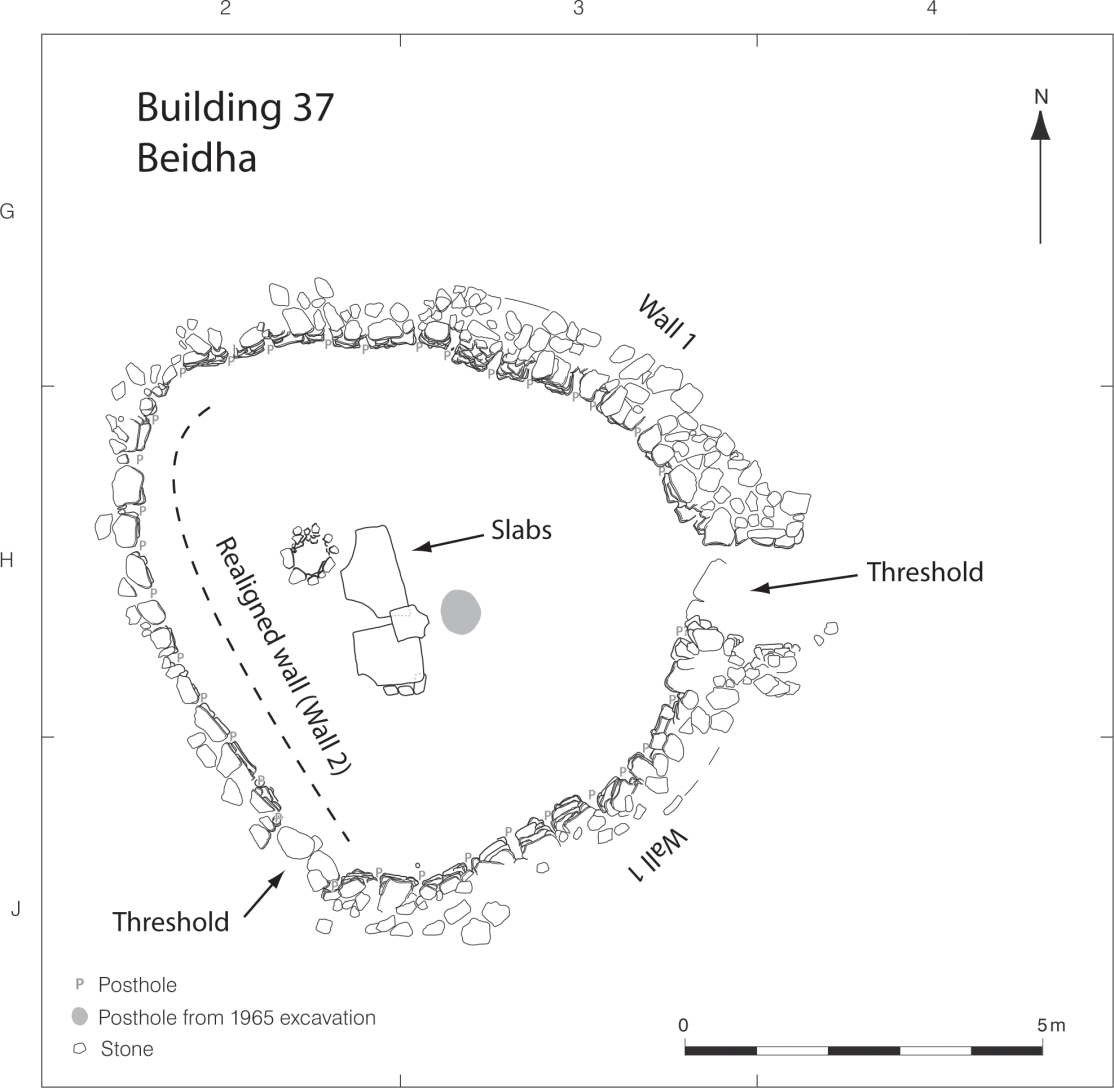
Horizontal plan of Building 37. Ground plan of new excavations indicating positioning of stone slabs, stone-lined posthole, and posthole identified during the Kirkbride excavations (grey circle). Arrows indicate contact between original Wall 1 and later addition Wall 2.

**Fig 6:**
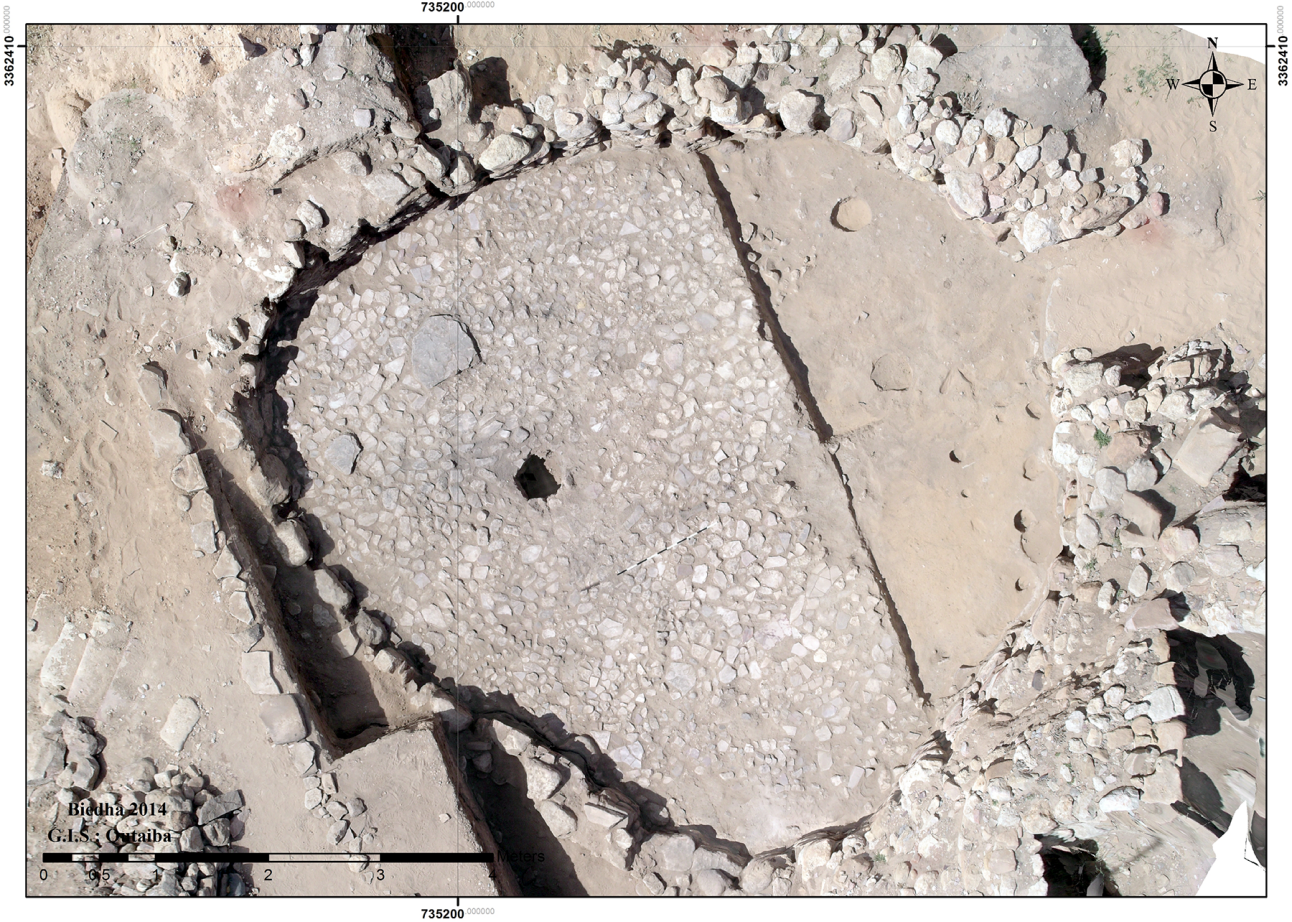
Sub-floor deposits in Building 37. Overhead view of Building 37 after removal of the clay floor surface (eastern half of structure) and sub-floor paving stones (western half of structure). Deposits and features visible under the sub-floor paving stones are pre-Building 37 and include packed ash layers, shallow pits, and several postholes. Note the close proximity of the wadi on the west side of the Building 37; sediments in between Wall 2 and the wadi edge are largely overburden from the original Kirkbride excavations. The linear arrangement of stones west of Wall 2 were placed to define the edge of the excavation trench for tourists. Further to the west, rectilinear stones are a modern stairway also for tourist use.

**Fig 7:**
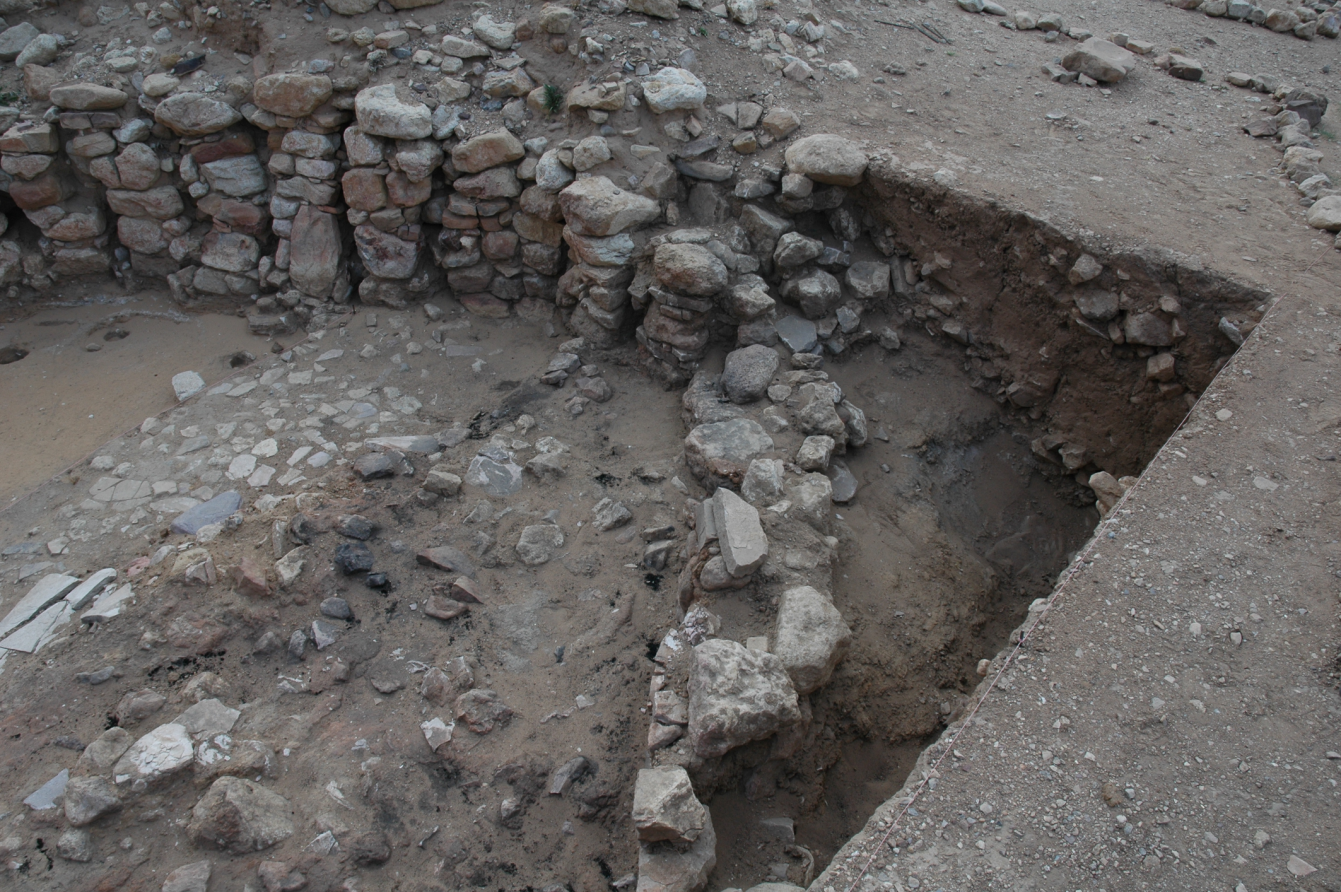
Wadi edge outside Wall 2. View of the steep slope into the wadi contemporary with Wall 2. The angle of slope makes it clear this wall is not in a semi-subterranean position, and is exposed to erosion.

**Fig 8:**
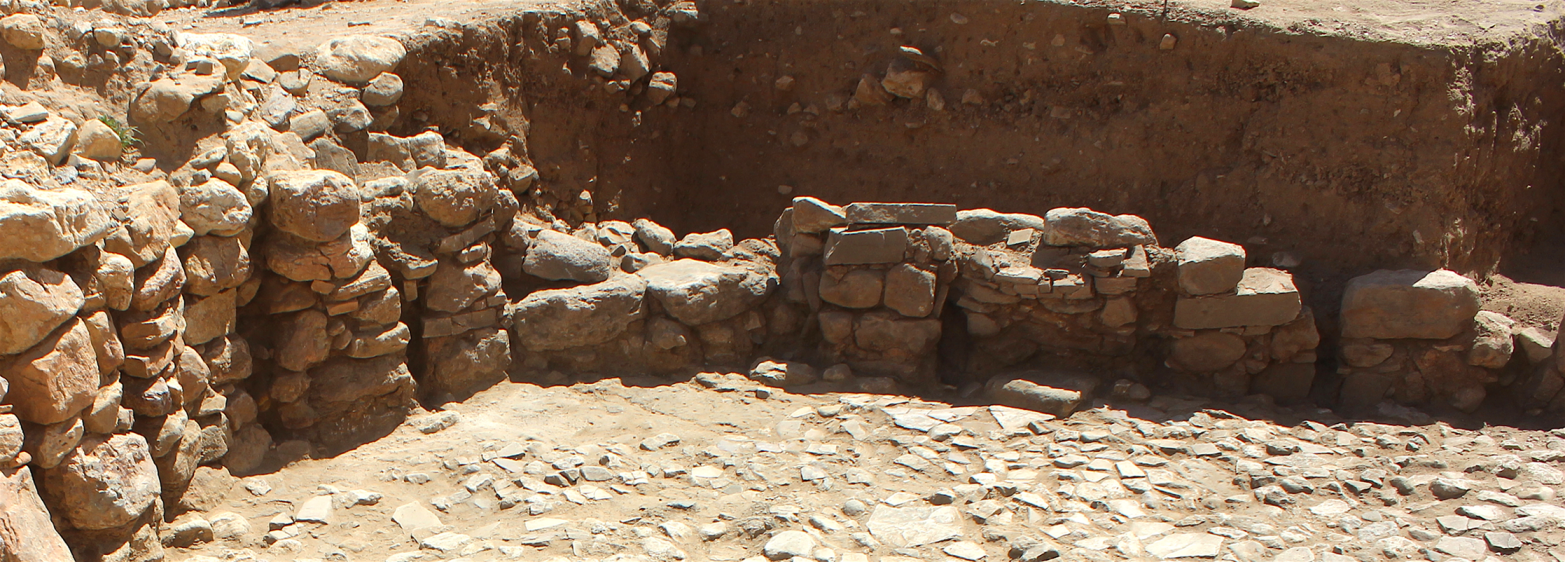
Building 37 entrance. Entrance into Building 37 located at the southernmost apex of Building 37 surviving as a threshold stone in a later phase of the structure wall.

Complete excavation of Building 37 revealed that the structure was not circular in shape as anticipated by Kirkbride (Fig 5). Instead, the renewed excavations indicate that that the original building ground plan was initially oval in shape, with its long axis running east-west, so the original interior area would have been considerably greater than initially assumed by Kirkbride (Fig 6). Moreover, the new excavations revealed that the western portion of the wall section was not part of the original Building 37 wall construction (Wall 1), but a later addition. This later wall (Wall 2) addition was constructed using the same methods used in the original wall and other Phase A domestic architecture (i.e., a thick stone rubble wall with post-slots present on its interior face), but unlike the original Wall 1, this later wall addition was not semi-subterranean. The later Wall 2 addition also sits on a different alignment to the original Wall 1. This is most clearly visible where Wall 2 springs off from the southern part of the original wall line at an angle much sharper than the expected curvature the original wall should have followed if it was to create a symmetrical elliptically-shaped building (Fig 5); the later wall addition meets the northern part of the original wall at a similarly sharp angle. Further evidence that Wall 2 was a later wall addition is found in the section profile that cross-cuts the southernmost edge of the original wall. Here, remnants of collapse originally from the western section of the original wall are visible; the rest of the original wall likely fell in to the eroding wadi (Fig 7). A second entryway and accompanying threshold stones were also identified in the southwestern portion of Wall 2; this entryway was not blocked (Fig 8).

Maintaining the size of Building 37 after the partial collapse of Wall 1 was clearly important. Even after the installation of Wall 2, the building was extremely large exhibiting a maximum north-south dimension of 6.5m, and east-west 7m, with an internal area of ca. 35m^2^. This is more than double the size of the next largest Phase A building and three times the mean interior area of all Phase A architecture (ca. 10m^2^).

However, the alignment of the new wall along the eroding wadi edge, while maximizing Building 37’s remaining interior space, left the wall exposed and risked continued collapse. The poor preservation of Wall 2, which is much reduced in height compared to the original wall and had also lost its exterior face, suggests the position of the later wall addition was quite precarious and, eventually, continued to tumble down the eroding wadi slope. The overall instability of the western portion of Building 37 is further attested by a series of crack lines and slumping visible within the westernmost portion of the clay floor surface (Fig 3), which was preserved when Building 37 was destroyed by fire and the floor surface baked after the burning roof collapsed.

Some (ca 4m^2^) later PPNB unexcavated deposits remain outside the south side of Building 37 and may conceal an additional Phase A building although this is unlikely given the small area of unexcavated deposits and absence of walls eroding out of the western, wadi-side edge of these deposits. The combination of open space to the east of Building 37, the wadi edge located immediately to the west of the structure, a lack of a space suitably large enough to accommodate an additional building between 41/56 and 37, and the likely absence a structure immediately south of Building 37, together suggests that Building 37 was set at least slightly apart from contemporaneous Phase A buildings. The relative isolation provided by the space around Building 37 is in marked contrast to the majority of the other Phase A buildings which are closely packed together, regularly share walls with one another, and often incorporate older Phase A walls into newer Phase A structures. The separation of Building 37 from other Phase A buildings appears intentional and designed to mark Building 37 as distinct (Fig 2). Communal buildings located in the northern Levant and dating to the earliest PPNB, for example at Jerf al Ahmar, show a similar isolation from contemporary structures [41].

## Discussion

### Mortuary practice and MPPNB community cohesion

In the southern Levant, elaborate mortuary practices involving the primary interment of single individuals under house floors, later removal of skulls in some cases, skull caching, and skull plastering emerged during the MPPNB and have been variously understood as evidence for household-based ancestor cults [42, 43, 44], emerging concepts of lineage and property [45], social differentiation [34], and social cohesive mechanisms that served to integrate increasingly autonomous households within the community in Pre-Pottery Neolithic societies [46]. Perhaps the most notable feature distinguishing MPPNB mortuary practice from earlier Neolithic ones is the number of secondary mortuary treatments applied to the dead and the scheduling of the rituals associated with each treatment [47, 34, 35]. Advance scheduling of multi-stage mortuary practice would have provided opportunities to coordinate the mortuary activities of disparate households and ensure maximum participation by community members [34]. Furthermore, the spacing of mortuary events would have facilitated manipulation of memory over the passage of time, as individuals were forgotten and transformed into communal ancestors, a process aided by the loss of individuality through the processes of plastering and decoration [33, 34, 35, 46, 48, 49]. Ultimately, this elaborate multi-stage mortuary process may have facilitated the cross-cutting of household lines to integrate communities [34, 35].

If primary burial and skull removal were employed to connect the living to the dead as individual ancestors within the household, secondary mortuary practices and the manipulation of memory served to bind households together within the wider community, over time transforming ancestral identities from the individual to the communal [35]. Symbolically charged plastered skulls that had been transformed into communal ancestors could have been used to create a shared experience, reaffirming the relationships within and between households and enabling these mortuary practices to serve as integrative mechanisms.

Shared experiences are generated in large part through active performance and by participation in groups events regardless of their precise form. MPPNB mortuary practices and associated rituals would have likely been low in frequency, but the combination of dramatic sensory equipment represented by the inherently charismatic plastered skulls, the large number of people and households drawn together by mortuary activities, and the occasional nature of such events, would have made for high-impact ritual gatherings [50]. These highly emotionally charged ceremonies facilitated, whether intended or not, community cohesion through the strong emotions and subsequent vivid memories that these gatherings generated [50]. The considerable investment and skills involved in preparing the skulls, and in the planning and scheduling of the ritual and ceremony, reveals a degree of elaboration and attention that suggests that these mortuary practices were sharply distinct from mundane activities [51]. Moreover, these mortuary practices were likely governed by highly-context specific social rules and modes of interaction that were embedded within the restricted frameworks of formally ritualized practices and, therefore, rarely enacted outside of such a mortuary context. Such high-impact rituals may have been dramatic and exciting, but as part of an emergent sacral world (e.g. [52]), those same practices that may have engendered the promotion of community cohesion in specific mortuary contexts were likely separate from those habituated elsewhere.

### Communal architecture and social integration

Middle PPNB mortuary routines involving skull removal, plastering, and the attendant social cohesive qualities they conferred on communities are generally understood as a southern Levantine phenomenon [35, 18]. However, the manipulation and circulation of crania were not practiced at Beidha, where all interred MPPNB individuals retained their skulls [20]. In contrast to the complex mortuary sequences practiced elsewhere during the MPPNB, the Phase A burials were not strongly marked by any distinct mortuary practice, having been simply laid out on accumulating rubbish, albeit sometimes on top of stone slabs. Inspection of original photographs suggests some human burials were placed on slabs, some of which appear to show signs of shaping ([27], Fig 242). While such burial treatment would undoubtedly have carried symbolic significance, they lacked the elaborate, formalized, and organized scheduled mortuary practices associated with the secondary treatment of the dead, in particular skull manipulation, seen elsewhere in the southern Levant and argued to be central to building community cohesion. The absence of scheduled mortuary ceremonies involving the curation, modification, display and handling of skulls that is thought to have brought community members together strongly suggests that the Beidha mortuary ritual was unlikely to have provided a strong social integrative mechanism. Instead, the built environment provided by structures such as Building 37, while having little association with formalized ritual practice, provided an alternative means to community cohesion through its close relationship with daily life.

The organization of the built environment draws from social, cultural and cosmological principles. Buildings do not passively provide the spaces for everyday life, but through their construction, modification, and use actively provide a means to create and manipulate those spaces for various social purposes [53]. As such, buildings are not a back-drop to life, but active constituents of society, framing routine behavior, social norms, memories, values and ideologies [54, 55]. The built environment can therefore shift in form and function over time, reflecting social dynamics and reinforcing changes within the community [56]. This can be seen in the numerous modifications often made to early PPN buildings in southern Jordan, and at Building 37 in Beidha with the replacement of the western entrance with an alcove and the addition of a new entrance, which would have changed not only the direction of access to the building, but closed it to the open courtyard to the west. Such modifications of physical structures take place within an ongoing context of everyday life where different people, or groups of people, use a space for different purposes at different times, changing its function by their actions. In turn, the social role of buildings shifts with the multiple rhythms of community life, planned and unplanned, festive and workday [54].

The relationship between the built environment and social organization is seen at Beidha in Building 37. The relatively large-scale construction seen here, involving the preparation of the building site, procurement of substantial quantities of stone, clay, sand, timber and water, and assembly of the building suggests a cooperative effort that involved a substantial portion of the Beidha community. Community-wide participation in this cooperative activity would have been straightforward and uncomplicated, as the skills and knowledge required to erect Building 37 –which replicated construction techniques utilized for domestic Phase A buildings, would have been familiar and routinely used. While numerous features are exclusive to Building 37, it is striking how the wall construction style, overall shape, lack of internal sub-divisions, and the presence of two entryways, one of which was subsequently blocked, all closely echo the construction design seen in Phase A domestic buildings. This shared grammar of design suggests that the activities featured in Building 37 were an extension of the range of mundane practices that took place in the much smaller buildings typical for the MPPNB settlement at Beidha. The practices could have ranged from several individuals undertaking their own tasks within a collective social space to tasks that require teamwork and the integrated knowledge and skill sets of several individuals.

The conventional construction of Building 37 and the absence of highly visible symbolic props (other than the enigmatic shaped stone slabs on the floor) conveys a lesser, or less formal, symbolic quality in the practices that took place within the building and suggests fundamental differences between the function of communal buildings and the occasional, more formalized, sacral roles played by mortuary practices. In contrast to MPPNB mortuary sequences practiced elsewhere in the southern Levant, the prosaic and routine activities practiced in Building 37 would likely have been high-frequency, albeit with a relatively low dramatic content. Routine, daily practices need not be highly ritualized, and their value arises from the generation of habitus through the ingrained routines embodied within the communal architecture [53]. In contrast to the punctuated rhythm of mortuary practice, interactions facilitated by communal architecture would have been regular components of everyday life transmitted through daily practice.

The overtly everyday character of Building 37 was countered by its physical detachment from the surrounding agglutinative architecture that distinguished it as a special building to sharpen focus on the communal aspects of the activities conducted there and the shared community-ethos they represent. The physical separation of Building 37 combined with the open space located outside its eastern entrance, would have also exposed movement in and out of Building 37 and signaled who was using the space, attending gatherings, and also the configuration of assembled groups, even if their actual activities were not always visible to the rest of the community.

Although Building 37 was likely used for occasional dramatic celebrations, the ease of access to the structure and evidence for heavy use suggests it was a space for routine and regular practices, interactions, and communication. The large open-floor plan, while probably insufficient to house the entire Beidha community at one time, still would have facilitated easy flow of movement and communication between individuals and small groups. These small-scale, regular and repetitive interactions operated through quotidian practices conducted within Building 37 would have fostered the production and reproduction of social norms to generate highly durable modes of community cohesion. This would have been in sharp contrast to the pathway of community integration promoted by skull plastering, which would have been directed by a suite of exceptionally context specific social interactions ordered by rules embedded within the symbolism and practice associated with mortuary ritual–not those that were part of daily practice. Their maintenance likely depended heavily on mnemonic triggers provided by the symbolic imagery used during infrequent mortuary rituals and the memories of those who attended previous rituals, while the routine engagement sustained by communal buildings themselves would have fostered an entirely different avenue of social integration. Unlike the highly ritualized and perhaps even hidden practices associated with the cycle of skull manipulations, especially those involved with the burial, caching and disguising skulls with plaster–a craft which likely entailed individual specialist, or even secret, knowledge of formal ritual rules, the shared knowledge of building maintenance would have provided a technical vocabulary that helped maintain community communication over the long-term.

Modifications to Building 37 not only served to preserve the building as the wadi edge eroded, but also suggest that its use evolved. While it remains unclear whether the new entrance created when the new wall (Wall 2) was constructed replaced an earlier entryway present in the collapsed original wall, or its insertion coincided with the blocking of the eastern entrance present in Wall 1, its construction confirms the relative ease with which stone structures could be modified. Blocking the eastern entrance transformed access to the structure, removing its direct connection and continuity into the open space to the east, but this apparent reduction in public accessibility may not have been the motivation for the shift, or even an intended consequence. Within the context of the social significance of the construction process, it may have been more important for the new builders to place their imprint on the structure with a new entrance, while paying respect to and reminding the community of the previous builders by creating the alcove as a reference to the location and opening for the old entrance. The long use of Building 37, shown by its rebuilding and repair, grounded in and echoing the routine repair and modification seen in contemporaneous Phase A houses could have provided multi-generational connectivity over time, transgressing time so that the building became an enduring locale and fixture of social connection and memory, critical to providing a sense of routine stability and maintaining a sense of community (c.f. [56]).

## Conclusions

Once completed, Building 37 would have served as a daily reminder of the cooperative work invested in the structure and, through this reminding, would have helped reinforce the social norms, values and ideologies that knitted the community together [53, 57, 58]. Furthermore, the ongoing maintenance activities required to sustain Building 37 long after its completion, especially those associated with keeping the roof watertight, would have provided an active medium that served to renew the memory of the initial building construction and associated social consonance while simultaneously maintaining and extending communication and cohesion between community members well beyond the initial building event. The rebuilding of the western portion of the Building 37 wall after its collapse into the wadi below further emphasizes the central and continuing role of this building in sustaining the fabric of the community, indeed, the process of redesign and rebuilding would have been important in itself to sustain the corporate learning and knowledge achieved in the initial construction.

Previously, ‘nondomestic’ buildings and the associated ritual activities that presumably took place within them were thought to function as a community regulatory mechanism that was vital for maintaining social cohesion within increasingly sedentary and populous communities progressively fragmented by the ascent of autonomous households and social inequality during the PPNB [6, 38, 59]. Instead, we propose that communal buildings, such as Building 37 at Beidha, did not emerge from new social stresses arising during the PPNB, nor were they related to novel ritual practices, but rather were part of a temporally deep ethos of community that emerged a thousand years earlier. The MPPNB communal structure at Beidha is rooted in PPNA traditions unique to southern Jordan, where communal architecture was commonplace and ranged in function from the highly mundane shared storage facilities at Dhra’ and WF16, to the participatory purpose of the large tiered structure at WF16, and the more visibly sacral role of the community mortuary structure at el-Hemmeh. This variability is continued in the MPPNB of southern Jordan, where Building F at Shkarat Msaied with multiple burials and one skull cache, appears to continue the tradition of communal mortuaries found at el-Hemmeh, while at Beidha, Building 37 facilitated regular, daily interaction between community members. This variability emphasizes that was more than one path to social integration during the PPN, even within southern Jordan, and that communal architecture was a crucial medium of social integration in nascent food producing societies.
